# Solid Oxide Cell Electrode Nanocomposites Fabricated by Inkjet Printing Infiltration of Ceria Scaffolds

**DOI:** 10.3390/nano11123435

**Published:** 2021-12-18

**Authors:** Simone Anelli, Luis Moreno-Sanabria, Federico Baiutti, Marc Torrell, Albert Tarancón

**Affiliations:** 1 Department of Advanced Materials for Energy, Catalonia Institute for Energy Research (IREC), Jardins de les Dones de Negre, 1, Sant Adrià de Besòs, 08930 Barcelona, Spain; sanelli@irec.cat (S.A.); fbaiutti@irec.cat (F.B.); mtorrell@irec.cat (M.T.); 2 Institute of Ceramics and Glass (ICV-CSIC), Kelsen 5, 28049 Madrid, Spain; l.moreno@icv.csic.es; 3 ICREA, Passeig Lluís Companys 23, 08010 Barcelona, Spain

**Keywords:** inkjet printing, solid oxide fuel cells, infiltration, mesoporous, oxygen electrode, electrochemistry

## Abstract

The enhancement of solid oxide cell (SOC) oxygen electrode performance through the generation of nanocomposite electrodes via infiltration using wet-chemistry processes has been widely studied in recent years. An efficient oxygen electrode consists of a porous backbone and an active catalyst, which should provide ionic conductivity, high catalytic activity and electronic conductivity. Inkjet printing is a versatile additive manufacturing technique, which can be used for reliable and homogeneous functionalization of SOC electrodes via infiltration for either small- or large-area devices. In this study, we implemented the utilization of an inkjet printer for the automatic functionalization of different gadolinium-doped ceria scaffolds, via infiltration with ethanol:water-based La_1−x_Sr_x_Co_1−y_Fe_y_O_3−*δ*_ (LSCF) ink. Scaffolds based on commercial and mesoporous Gd-doped ceria (CGO) powders were used to demonstrate the versatility of inkjet printing as an infiltration technique. Using yttrium-stabilized zirconia (YSZ) commercial electrolytes, symmetrical LSCF/LSCF–CGO/YSZ/LSCF–CGO/LSCF cells were fabricated via infiltration and characterized by SEM-EDX, XRD and EIS. Microstructural analysis demonstrated the feasibility and reproducibility of the process. Electrochemical characterization lead to an *ASR* value of ≈1.2 Ω cm^2^ at 750 °C, in the case of nanosized rare earth-doped ceria scaffolds, with the electrode contributing ≈0.18 Ω cm^2^. These results demonstrate the feasibility of inkjet printing as an infiltration technique for SOC fabrication.

## 1. Introduction

Solid oxide cells (SOCs) are devices which can reversibly produce and utilize hydrogen for chemical to electrical energy conversion with high efficiency [1,2,3,4,5,6]. State-of-the-art (SoA) materials for SOCs are ionic conductors such as yttrium-stabilized zirconia (YSZ) and Gd-doped ceria (CGO) for the electrolyte, Ni-YSZ ceramic metallic composites for the fuel electrode and mixed ionic–electronic conductors (MIECs) such as La_1−x_Sr_x_Co_1−y_Fe_y_O_3−*δ*_ (LSCF) for the oxygen electrode [7]. Composite electrodes involving MIEC perovskites together with pure ionic conductors (e.g., YSZ and CGO) improve the performance of the cell, increasing the active region of the electrode called the triple phase boundary (TPB). The TPB is the region where ions, electrons and the gas are in contact and where oxygen reduction reactions (ORRs) take place during operation in the fuel cell mode [8,9,10]. Although employing an electrolyte material in electrode composites increases chemical and thermo-mechanical compatibility, the required high-temperature treatments present some drawbacks. One of the most important is the formation of insulating secondary phases between SoA strontium-rich perovskites and zirconia-based electrolytes (i.e., La_2_Zr_2_O_7_ and SrZrO_3_) [11,12,13]. To improve compatibility, barrier layers of ceria are commonly introduced between the electrolyte and the oxygen electrode [14,15]. Barrier layers demonstrate the ability to grant great stability at the electrolyte–electrode interface during high-temperature fabrication and operation [14]. Moreover, low-temperature synthesis of infiltrated perovskites inside a pre-sintered electrolyte scaffold, which substantially further reduces the formation of unwanted phases, has also been recently demonstrated to be particularly effective [16,17,18]. Infiltration typically requires low amounts of active material (metal–salt solutions [19], nanoparticles in suspension [17] or molten salts [20]), resulting in well-distributed nanosized and nanostructured electrodes [18]. To ensure proper dispersion of the solution, the use of different additives such as urea, citric acid, glycine, and ethylene glycol [19] is required. The use of pre-sintered scaffolds with ordered structure and small porosity can promote the fabrication of functional layers with high active areas and proper distribution of the infiltration solution. In particular, nanocomposites based on mesoporous ceria backbones demonstrate excellent performance as electrodes for SOC applications [21,22,23,24]. Mesoporous materials are characterized by small porosity (2–50 nm in diameter), a high surface area (>100 m^2^ g^−1^) [25,26,27], and good percolation pathways, which mitigate problems associated with local high current densities in SOC operation [28,29,30,31,32,33,34,35,36,37]. The synthesis of mesoporous materials typically involves the impregnation of sacrificial templates such as SiO_2_ that, after calcination, are removed by chemical etching. In particular, nanocasting using hard templates allows the fabrication of powders with high ordered structures and complex lattices such as KIT-6 or SBA-15 [32,38,39,40,41].

Despite the above-mentioned advantages, infiltration is currently carried out manually with a micro-pipette [19,37,42,43,44,45,46,47,48,49] or via dip coating [50,51], compromising its reproducibility, robustness and scalability. The implementation of additive manufacturing (AM) techniques for the automation of infiltration steps, in particular using inkjet printing (IJP), is a possible solution to standardize the process [52,53,54,55,56,57,58]. IJP is a high-resolution printing technique where small droplets of ink (with a volume in the range of 1 nL^−1^ pL) are deposited on a substrate. In standard domestic IJP applications, black or cyan, yellow, magenta, black (CYMK) inks are deposited on paper but the technology has been broadly extended and it is now a very versatile technique. IJP is used for the deposition of submicronic particles (e.g., precious metal and ceramic materials) [59,60,61] in electronics [62], rapid prototyping [63], ceramic-based components [59,60] and sensors [64]. The viscosity range for ceramics-based inks for IJP is 1–20 mPa s and the deposited layer thickness is from below 100 nm up to 100 μm [65,66]. This, together with the low solid loading of the inks (1–10 vol%), makes IJP a time-consuming technique that is more suitable for low aspect ratios or 2D objects (opposite to other AM techniques that are more adequate for the fabrication of high-aspect ratio 3D parts) [67]. IJP is broadly classified as either continuous inkjet (CIJ) [59,68,69] or drop-on-demand (DOD) [59,70,71] printing. CIJ consists of a pressurized flow of ink which generates a continuous stream of drops through a micro-scaled nozzle. When printing is not required, the stream is deflected to a gutter for recycling [59]. On the other hand, DOD-IJP is based on a stream of droplets generated only when required for printing (avoiding unnecessary waste of material) [60,71]. Drop formation requires a pressurized liquid which generates droplets driven by Ryleigh instability.

DOD-IJP has been used in the field of SOCs to produce different components, from dense electrolytes [72] to composite electrodes by infiltration [56,57]. Typical inks used for DOD-IJP deposition of ceramic materials are colloidal suspension and sol–gel solutions. Such inks are usually characterized by densities of approximately ≈1 g cm^−3^, viscosity in the range of 1–30 mPa s and surface tension in the range of 35–70 mN m^−1^ [73]. Optimal rheology of the inks is essential to avoid clogging of the nozzles, the formation of satellite drops [74] or splashing [61] of the liquid on the substrate. In the literature, the Reynolds (*Re*) and Weber (*We*) numbers are used to describe the properties of inks [59,75] either alone or combined together in the so-called Ohnesorge (*Oh*) number [59]. The most commonly employed rheological parameter is the inverse of the *Oh* number, called *Z*, and it is calculated following the next expression (Equation (1)) [71]:

(1) $${Z=Oh}^{-1}=\frac{Re}{\sqrt{\;We}}=\frac{\sqrt{\gamma \rho a}}{\eta }$$

where *a* is the average diameter of the droplet (which corresponds to the nozzle diameter) and *ρ*, *η* and *γ* are the density, the dynamic viscosity, and the surface tension of the ink, respectively. *Z* is a widely employed and reliable parameter because it is independent of the jetting speed and can be experimentally determined. Suitable printability ranges are usually accepted for 1 ≤ *Z* ≤ 10 [76,77,78] and 1 ≤ *Z* ≤ 20 [71], although the upper limit is not totally clear, since stable printing has even been reported at *Z* >> 10 [79,80], e.g., *Z* = 100 [81,82,83].

In this work, automatic infiltration of electrolyte scaffolds with active electrode materials is employed for the fabrication of functional layers in solid oxide cells. In particular, DOD-IJP is used to infiltrate mesoporous ceria-based pre-sintered backbones with LSCF. The performance of these composites is evaluated as oxygen electrodes in symmetrical cells consisting of YSZ/LSCF–CGO/LSCF. The produced cells were structurally, microstructurally and electrochemically characterized between 600 and 900 °C. Overall, the main goal of this study is to demonstrate the feasibility of IJP as an automatic infiltration technique for SOC application and to standardize the process for future application in large-area devices.

## 2. Materials and Methods

### 2.1. Formulation of LSCF Inks

To obtain a La_0.6_Sr_0.4_Co_0.2_Fe_0.8_O_3_ perovskite, a solution 0.1 M of La(NO_3_)_3_·6H_2_O, Sr(NO_3_)_2_, Co(NO_3_)_2_·6H_2_O and Fe(NO_3_)_3_·9H_2_O (Merck, Darmstadt, Germany) was prepared in stoichiometric proportion. Polyvinylpyrrolidone (PVP—Merck, Darmstadt, Germany) the dispersant and glycine (C_2_H_5_NO_2_—Merck, Darmstadt, Germany) as the complexing agent were dissolved in an ethanol:water (70:30 vol%) medium under continuous stirring at room temperature. After the complete dissolution of PVP and glycine, the precursors were added one by one, under continuous stirring. The formulation of the LSCF sol–gel solution was optimized for handmade infiltration in previous works of the group to achieve good permeability inside the ceramic backbone [35,36,37].

### 2.2. Rheological Characterization

A DV3T Rheometer (Brookfield Engineering Labs., Inc., 11 Commerce Boulevard, Middleboro, MA, USA) was used to measure the viscosity of the produced ink. The instrument can measure the viscosity of a liquid medium at given share rates. It has a speed rate which from 0.01 to 250 RPM, a viscosity accuracy of ±1.0% and a viscosity repeatability of approximately ±0.2%.

Surface tension measurement of the ink was made by an OCA20 optical contact angle and contour analysis system (DataPhysics Instruments GmbH, Filderstadt, Germany), using the pendant drop method. This method can determine the surface tension (*σ*) of a liquid medium from the Young–Laplace equation (Equation (2)).

(2) $$\Delta p=\sigma \;\left(\frac{1}{r_{1}}+\frac{1}{r_{2}}\right)$$

In the equation, Δ*p* is the difference in pressure between the two sides of the curved surface and *r*_1_ and *r*_2_ are the two curvature radii of the drop.

### 2.3. Symmetrical Cells Fabrication

Symmetrical electrolyte-supported cells were prepared using tape-casted 8YSZ (FAE S.A.U., L’Hospitalet de Llobregat, Barcelona, Spain) as electrolyte and cell support with a diameter of ≈2 cm and a thickness of ≈150 µm. A roughness promoter layer was sprayed on top of this this dense YSZ layer by 3-axis automated airbrushing (Print3D Solutions, Albacete, Spain) using ethanol-based ink with commercial Ce_0.8_Gd_0.2_O_1.9_ powders (Kceracell, Geumsan, Korea) as the solid load. The coating was deposited on both sides of the YSZ electrolyte and sintered at 1250 °C for 3 h. This layer was deposited in order to increase the roughness of the surface and to improve the attachment of the electrodes to the electrolyte, minimizing the contact resistance of the interfaces. A barrier layer of CGO, decorated with 5 wt% of Co oxide as sintering aid, was deposited on top of the roughness promoter layer to avoid reactivity between the YSZ and the LSCF layers. The barrier layer (≈15 μm) was sintered at 1275 °C for 2 h (2 °C min^−1^) before the deposition of the oxygen electrode [14]. Then, the different CGO scaffold layers (thickness ≈60 µm) were equally deposited on top using the same method. The composition of the inks for CGO airbrushing is detailed elsewhere [35]. Three cells were fabricated with commercial CGO scaffolds on both sides and sintered at 1250 °C for 2 h and one cell was prepared with mesoporous scaffolds on both sides and sintered at 900 °C for 5 h. Mesoporous Ce_0.8_Gd_0.2_O_1.9_ powder employed in the CGO scaffold was synthesized as detailed elsewhere [28,33,34]. The obtained active area of the electrodes was ≈1.54 cm^2^ (≈1.4 cm diameter) in all cases.

The infiltration of the ceria scaffolds was automatically conducted by inkjet printing, using a customized printer, produced by Print3D Solutions (Albacete, Spain), which uses a commercial cartridge C6602A from Hewlett-Packard (Palo Alto, CA, USA) with a nozzle diameter ≈60 µm. The movement of the 3-axis system is controlled by Arduino (https://www.arduino.cc/, webpage consulted the 13 September 2021) and the printing process (with the C6602A cartridge) by Processing© (version 3.1.1, Processing Foundation, Brooklyn, NY, USA). The printing system (Figure S1) allows modification of the saturation parameter of the ink from 1 to 20. The saturation parameter corresponds to the distance between a deposited droplet and the next one, and therefore this is proportional to the amount of ink deposited in a single step [84]. After a first optimization with different saturation parameters, poor control of the process was observed for high saturation (i.e., 15/20 sat. and 20/20 sat.). Conversely, good injection was observed for lover values such as 5/20 sat. and 10/20 sat., and therefore the latter values were chosen as parameters for the functionalization of symmetrical scaffolds in the present study.

Using the described methodology, four symmetrical cells were fabricated and characterized. First, a reference cell without any infiltration (CGO) was fabricated and measured while another two were infiltrated keeping ink saturation at 5/20 sat. (5-CGO) and 10/20 sat. (10-CGO), respectively. Finally, a mesoporous CGO symmetrical cell was infiltrated with saturation at 10/20 sat. (10-CGO_meso_). After infiltration, all the cells showed a total increase in mass of 25 ± 2 mg. Infiltration in all cases was separated in three different steps with a calcination treatment after each one at 500 °C for 30 min and a final thermal treatment at 800 °C for 3 h. The distance of the printing nozzles from the samples was ≈1 mm for all depositions. Since the 5-CGO cell was infiltrated with a lower saturation parameter (5/20 sat.) more depositions were necessary for each step in order to reach the same amount of infiltration solution in weight. In this manuscript, this deposition process is often referred as the “slow deposition process”, and the process with saturation at 10/20 sat. is referred to as the “fast deposition process”. The characteristics of the four different symmetrical test cells are reported in Table 1. A layer of commercial LSCF (Kceracell, Geumsan, Korea) powder was deposited on top of the scaffold of the four cells by airbrushing as current collector and sintered at 900 °C for 5 h.

### 2.4. Microstructural Characterization

Initial microstructural characterization of the obtained powders and cells was performed using a Carl ZEISS (Oberkochen, Germany) Auriga scanning electron microscope (SEM) equipped with an energy dispersive X-ray spectroscopy (EDX—X-Max, Oxford Instrument, Abingdon, UK) detector. The SEM-EDX characterization of the cells was conducted after electrochemical analysis. The crystalline phases were characterized by X-ray diffraction (XRD) *θ*–2*θ* (20 to 90°) measurements on a Bruker-D8 Advance equipment (Billerica, MA, USA) at room temperature using Cu-*K*_α_ radiation with a nickel filter and Lynx Eye detector. The mesoporous powders were also characterized using a Tristar II Brunauer–Emmett–Teller (BET) analyzer (Micromeritics, Norcross, GA, USA).

### 2.5. Electrochemical Characterization of Symmetrical Cells

Symmetrical cells were electrochemically characterized in a commercial ProboStatTM (NorECS AS, Oslo, Norway) station, inside a high-temperature vertical tubular furnace via impedance spectroscopy measurement (EIS—Novocontrol spectrometer by NOVOCONTROL Technologies GmbH & Co. KG, Bad Wildbad Baden Württemberg, Germany) from 900 to 600 °C (50 °C steps) and at synthetic air atmosphere. Gold paste (Fuelcellmaterials, Lewis Center, OH, USA) and meshes (Fiaxell Sarl, Lausanne, Switzerland) were used to ensure the current collection. The impedance measurements were conducted in potentiostatic mode from 10 to 100 mHz and an amplitude of 50 mV. Zview software (version 2.1, Southern Pines, NC, USA) was used to fit the impedance spectra.

## 3. Results and Discussion

### 3.1. Fabrication of the Symmetrical Cells

**Figure 1:**
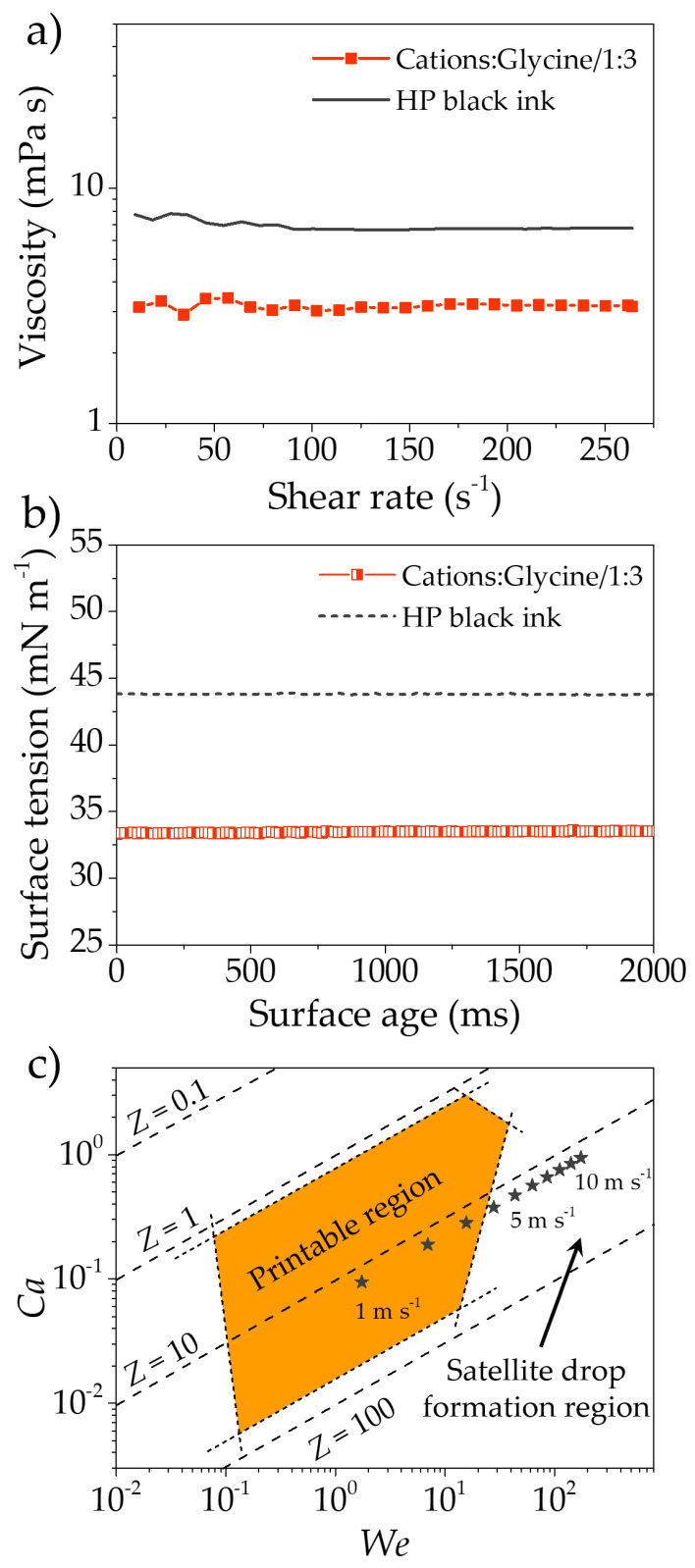
Viscosity (**a**) and surface tension (**b**) measurements of the sol-gel ink compared with the HP ink inside the cartridges [86]. (**c**) Printable region for IPJ deposition. The black stars represent the formulated ink considering the velocity range of the drops (1–10 m s^−1^). The ink is between the preferable region for jetting and the satellite drop region. Panel (**c**) was adapted with permission from H. C. Nallan, J. A. Sadie, R. Kitsomboonloha, S. K. Volkman, and V. Subramanian, Langmuir 30, 13470 (2014). Copyright 2014 American Chemical Society.

To ensure proper infiltration of LSCF inside the CGO scaffolds, the printability of formulated inks was evaluated. More specifically, the viscosity (Figure 1a) and the surface tension (Figure 1b) of the LSCF ink were measured to quantitatively determine suitability for inkjet printing [59,71,85]. After preliminary optimization (not showed here for the sake of clarity), LSCF infiltration ink presents a viscosity of 3.2 ± 0.1 mPa s and a surface tension of 33.1 ± 0.3 mN m^−1^. The viscosity shows typical Newtonian behavior, maintaining a constant value for the explored share rate range. The measured surface tension is very stable with time, which means that the evaporation rate of the solution is slow. The resulting *Z* number was calculated as 14.0 ± 0.4. This value of *Z* indicates good printability as recently discussed by Liu and Derby, who fixed the printability region at 1 < *Z* < 20 [71]. Further, Figure 1c shows the *We–Ca* plot (*We* number vs. the capillarity, *Ca*, of the suspension [86]) of the same ink, to provide a more exhaustive representation of its features. Since the jetting speed of the droplets produced by the HP C66002 could not be measured, *We* and *Ca* number ranges were estimated considering at a reasonable speed range from 1 to 10 m s^−1^ (black stars in the plot) [61,87]. It can be observed that at the considered speed range, the formulated LSCF ink overlaps the “printable” and the “satellite drops formation” areas. The formation of satellite drops is not considered an issue for the specific application of the infiltration and, during the printing process, no jetting issues were observed, experimentally confirming that the ink has good printability.

**Figure 2:**
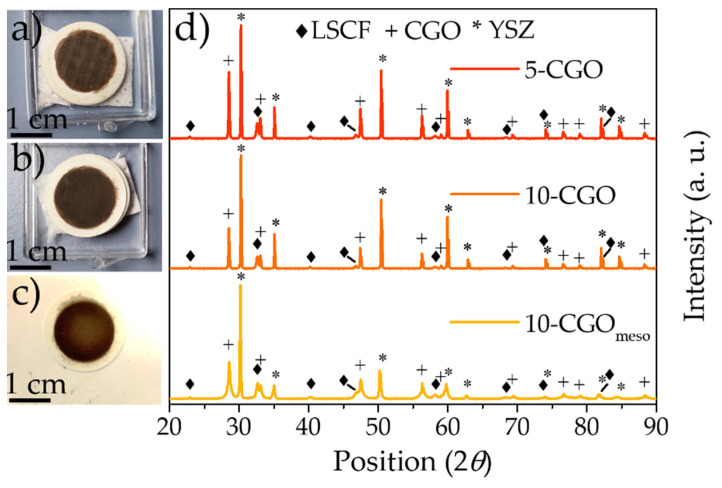
Digital pictures of the 5-CGO (**a**), 10-CGO (**b**) and 10-CGO_meso_ (**c**) cells. XRD of the 5-CGO,10-CGO and 10-CGO_meso_ cells after the infiltration and calcination of the LSCF solution (**d**). The presence of the LSCF characteristic peaks confirms the formation of the perovskite after the calcination process.

After optimization, the LSFC infiltration ink was printed on the pre-sintered CGO scaffolds. Then, the electrode layer was calcined at a low temperature (800 °C for 2 h) to crystallize the desired LSCF phase. Figure 2a–c show pictures of 5-CGO, 10-CGO and 10-CGO_meso_ samples (after calcination). The deposited layer of the 5-CGO cell seems generally less homogeneous compared with that of 10-CGO and of 10-CGO_meso_, which conversely demonstrate reasonable shaping and good homogeneity of the printed layer. XRD of the infiltrated electrode layers was carried out to investigate the crystallization and the reactivity of the LSCF perovskite. Figure 2d shows the XRD patterns of the 5-CGO, 10-CGO and 10-CGO_meso_ samples. All the observed reflections can be fully indexed with the YSZ, CGO and LSCF diffraction patterns, indicating the formation of the desired perovskite phase and the absence of secondary phases [88,89,90].

**Figure 3:**
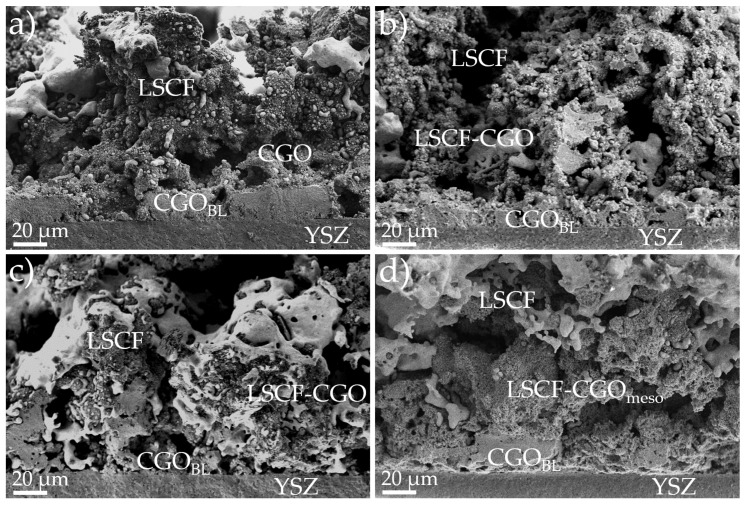
Cross-sectional SEM micrographs of the four different cells. (**a**) Micrograph of the CGO cell, fabricated with no infiltration. (**b**) Micrograph of the cell fabricated with slow infiltration and 5/20 sat. (5-CGO). (**c**) Micrograph of the cell fabricated with fast infiltration and 10/20 sat. (10-CGO). (**d**) Micrograph of the cell fabricated with a mesoporous CGO scaffold and fast infiltration (10-CGO_meso_).

Microstructural characterization of the electrode cross sections was achieved through SEM on the four cells, after the electrochemical analysis of the following section, to observe the difference in morphology due to the variations in infiltration. Figure 3 shows SEM micrographs of the four cells presenting the general microstructure features of the obtained electrodes. Observing the micrographs one can immediately recognize the common structure for the CGO (Figure 3a), 5-CGO (Figure 3b), 10-CGO (Figure 3c) and 10-CGO_meso_ (Figure 3d) cells. This structure consists of a YSZ electrolyte, a CGO barrier layer, a CGO scaffold infiltrated by LSCF in the case of 5-CGO, 10-CGO and 10-CGO_meso_, and a LSCF layer airbrushed with commercial powder. A top layer of gold paste used as a current collector during electrochemical characterization, presented in the following section of the manuscript, can also be observed. The four electrodes present good attachment to the CGO barrier layer. Despite the good results of PLD barrier layers proposed by Morales et al. [15], the sprayed CGO barrier layers presented in this work show lower densification upon sintering. A certain level of porosity is typical for the current SoA deposition techniques (i.e., spray coating and screen printing) [14]. However, the densification of the layer does not represent an issue in the case of the present study, where no long-term characterization was conducted and a low fabrication temperature (900 °C) is applied. Generally, the four electrodes present a similar structure with good porosity that allows LSCF infiltration to impregnate the CGO scaffold.

**Figure 4:**
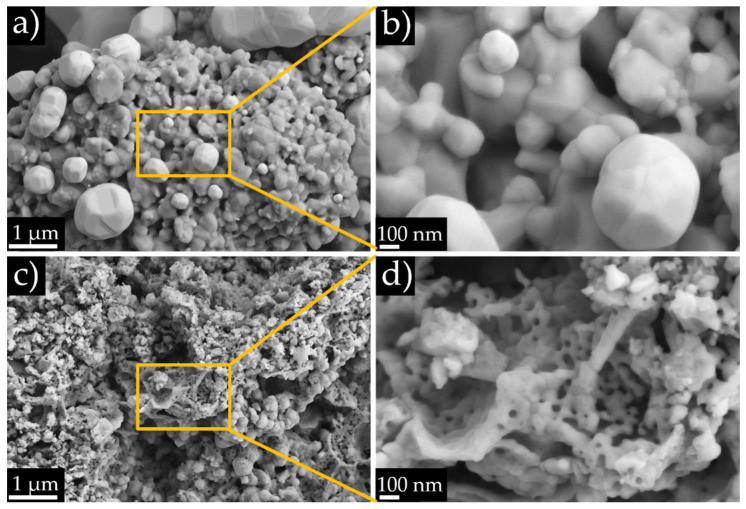
Representative region of the scaffold of the 10-CGO cell (**a**). SEM micrograph at a higher magnification (**b**). Representative region of the infiltrated scaffold of the 10-CGO_meso_ cell (**c**). SEM micrograph at a higher magnification (**d**) where the residual mesoporosity can be observed.

Figure 4 shows SEM micrographs at a high magnification of 10-CGO (Figure 4a,b) and 10-CGO_meso_ (Figure 4c,d) cells. The 10-CGO cell shows well-sintered coarsened particles with a reasonable but low porosity, while the scaffold of the 10-CGO_meso_ retains the presence of the mesoporous structure even after thermal treatment (although one can notice a clear evolution of the ordered structure during the thermal treatment with respect to the synthesized mesoporous powders shown in Supplementary Info Figure S2). These microstructural variations are mainly due to the nature of the original powder and the different sintering temperature of both 10-CGO and 10-CGO_meso_, which were treated at 1250 and 900 °C, respectively [33,91]. Because of the presence of the residual mesoporosity, the scaffold of the 10-CGO_meso_ cell offers more active area for chemical reactions. This, combined with the infiltration of the LSCF phase is expected to increase the TPB of the electrode. Moreover, the interconnected mesoporosity should improve the gas distribution and the thermal distribution of the functionalized electrode [31].

**Figure 5:**
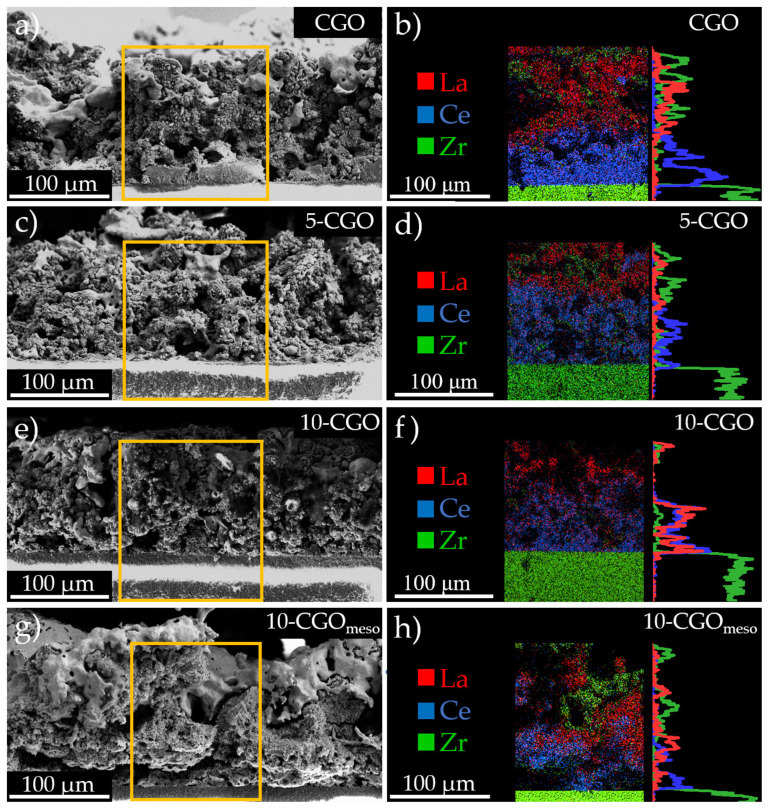
Representative SEM micrographs and EDX elemental maps of the CGO cell (panels (**a**,**b**)), the 5-CGO cell (panels (**c**,**d**)), the 10-CGO cell (panels (**e**,**f**)) and the 10-CGO_meso_ cell (panels (**g**,**h**). In all the EDX maps, the YSZ, CGO and LSCF phases are indicated by their main representative element. Zr (green), Ce (blue) and La (red) show the presence of YSZ, CGO and LSCF, respectively.

Figure 5 shows SEM images of the reference (CGO) and infiltrated cells (5-CGO, 10-CGO and 10-CGO_meso_). Regarding chemical composition, an SEM-EDX map of the reference cell (without infiltration) is presented in Figure 5a,b showing clear separation between the CGO scaffold (Ce signal in blue) and the LSCF current collector (La signal in red). A similar pattern is observed for 5-CGO in Figure 5c,d, suggesting poor infiltration of LSCF inside the CGO backbone under this infiltration condition. The amount of infiltrated LSCF clearly increases for 10-CGO and 10-CGO_meso_ (Figure 5e–h). This means that faster infiltration with higher saturation is highly beneficial even for mesoporous scaffolds. Although the total amount of deposited material is similar in all cases (see Experimental section), it has been observed that the slower deposition process (made in more steps with drying at room temperature after each one) likely causes the progressive obstruction of the top part of the scaffold, blocking the infiltration in subsequent steps. This can be well observed analyzing the obtained linescans, which show very little LSCF in the region of the CGO scaffold of the 5-CGO cell, especially close to the electrolyte–electrode interface, where the ORRs are required. Conversely, good penetration of the LSCF phase inside the CGO scaffold in the case of the 10-CGO and 10-CGO_meso_ cells with the fast process has been demonstrated. These results show better functionalization of the scaffold compared with traditional infiltration via micropipette injection reported in previous work of the group [35,37].

### 3.2. Electrochemical Characterization of the Infiltrated Cells

**Figure 6:**
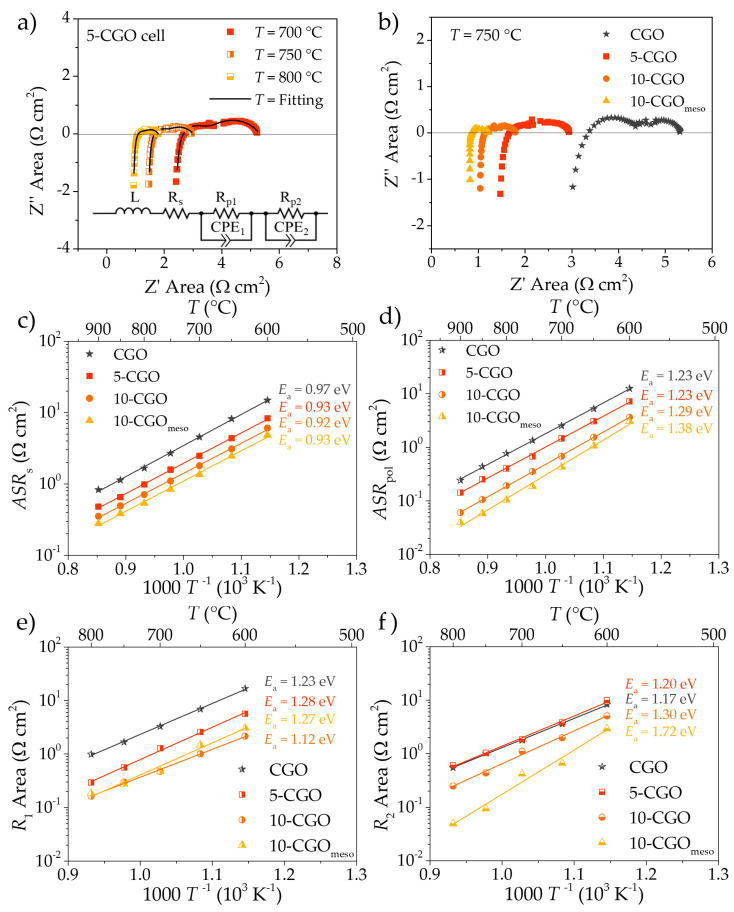
EIS spectra of the 5-CGO cell at 700, 750 and 800 °C. (**a**) The equivalent circuit used for the (**b**) EIS spectra of the four cells (CGO, 5-CGO, 10-CGO and 10-CGO_meso_) at 750 °C. (**c**) Serial *ASR* contributions of CGO, 5-CGO, 10-CGO and 10-CGO_meso_ cells as a function of the inverse of temperature. (**d**) Polarization *ASR* contributions of CGO, 5-CGO, 10-CGO and 10-CGO_meso_ cells as a function of the inverse of temperature. The values were obtained by fitting with the equivalent circuit shown in (**a**). (**e**) *R*_1_ and (**f**) *R*_2_ of the two ZARC elements used for the fitting of the EIS spectra as a function of the inverse of temperature.

The complete set of samples (CGO, 5-CGO, 10-CGO and 10-CGO_meso_) were characterized under symmetrical configuration by electrochemical impedance spectroscopy (Figure 6). Figure 6a shows the resulting impedance spectra of the reference cell 5-CGO tested at three different temperatures, 700, 750 and 800 °C, which are the typical operating temperatures for SOC devices with similar electrodes. Each impedance spectrum was successfully fitted applying the equivalent circuit shown in Figure 6a. This circuit, the simplest circuit able to fit the asymmetric impedance spectra, is composed by an inductance *L*, a serial resistance *R*_s_ and two ZARC elements. *R_s_* can be easily assigned to resistance contributions in series, mainly dominated by the electrolyte, while the combination of the resistance of the ZARC elements gives the total polarization resistance associated with the symmetrical electrodes (*R_pol_* = *R*_1_ + *R*_2_). In order to compare samples with different levels of infiltration, the impedance spectra of the four cells at a fixed temperature of 750 °C were plotted in Figure 6b. One can immediately notice the difference in terms of overall area-specific resistance (*ASR* = (*R_s_* + *R_pol_*) × *A*, where *A* is the area of the cell) of the four cells due to infiltration. The reference CGO cell presents an overall *ASR* of ≈5.3 Ω cm^2^, while a progressive decrease in this value is observed as a function of the deeper functionalization of the scaffold. The obtained *ASR* values for 5-CGO, 10-CGO and 10-CGO_meso_ samples were ≈2.9, ≈1.8 and ≈1.2 Ω cm^2^, respectively. Slower infiltration of LSCF inside the scaffold (5-CGO) reduces to almost half the overall resistance, compared to the reference value for the CGO cell. Faster infiltrated samples (10-CGO) divide the overall resistance of the reference value by a factor of three or four (with or without the mesoporous scaffold, respectively). The 10-CGO_meso_ cell presents the lowest measured *ASR*, ≈1.2 Ω cm^2^ at 750 °C. Considering electrodes with a similar composition, Sanna et al. reported an *ASR* of ≈2.8 Ω cm^2^ at the same temperature [92]. Conversely, Nielsen et al. reported an *ASR* of ≈0.34 Ω cm^2^ at the same temperature [93]. Considering only the contribution of the LSCF–CGO electrode (*ASR*_pol_) of the 10-CGO_meso_ cell, the value decreases to ≈0.18 Ω cm^2^, much closer to the 0.1 Ω cm^2^ measured by Nielsen et al. for their LSCF–CGO composite electrode [93]. The considerable ohmic contribution (*ASR*_s_) observed even for 10-CGO_meso_ cell (≈0.8 Ω cm^2^) could also be due to the airbrushed CGO barrier layer, deposited on top of the YSZ electrode in order to avoid the formation of secondary phases with the Sr-rich perovskite during the sintering step. Localized barrier layer discontinuities are reported in the microstructural characterization section (Figure 3). As mentioned earlier, previous works of the group pointed out the better efficiency and the good morphology granted by alternative deposition techniques such as PLD for CGO barrier layers [15,36].

The plots of serial and polarization *ASR* as a function of the inverse of temperature are reported at between 600 and 900 °C in Figure 6c,d. Slightly higher activation energy values of the serial resistance are obtained for the CGO cell (0.97 ± 0.04 eV) compared to those measured for 5-CGO, 10-CGO and 10-CGO_meso_ (0.93 ± 0.01, 0.92 ± 0.01 and 0.93 ± 0.02 eV, respectively). Despite both values being very close to that reported for the 8YSZ electrolyte [94], observed differences could be explained considering contributions to the serial resistance of both the electrolyte and the CGO scaffold [44,95]. The observed reduction in serial *ASR* values is likely related to a decrease in the CGO layer on the better infiltrated functional layers that improves the current collection of the cells (promoted by the homogeneous and continuous infiltration of the LSCF phase through the CGO scaffold).

Regarding the study of polarization resistance (Figure 6d), the activation energy range is from 1.2 to 1.4 eV, which is typical for LSCF–CGO composites previously reported in the literature [54]. Cells with a commercial CGO scaffold present similar values (1.23 ± 0.01, 1.23 ± 0.02 and 1.29 ± 0.01 eV for CGO, 5-CGO and 10-CGO cells, respectively) while a slight increase is observed employing the mesoporous scaffold (1.38 ± 0.04 eV). This difference could be caused by either the presence of small amounts of silica contamination (≈1 wt%), due to the incomplete removal of the employed mesoporous template, or dopant segregation to the interfaces, as previously reported by the authors [36,96]. Concerning *ASR*_pol_ values, a clear difference in resistance between scaffolds with and without infiltration is observed (due to the lack of a catalytic phase in the functional layer). Moreover, an increasing beneficial effect of infiltration is observed for higher saturation and the use of mesoporous CGO scaffolds, which implies a higher active area. Better LSCF distribution within the scaffold for 10-CGO (see Figure 5f,h) and the considerable increase in active area in 10-CGO_meso_ (see Figure 4d) are straightforward arguments that justify better performance by extension of the TPB active region of the functional layer. Overall, as in the case of serial resistance, the combination of better infiltration a mesoporous microstructure improves the performance of the electrode significantly.

Figure 6e,f show the plots of the two contributions to the *ASR*_pol_ (*R*_1_ and of *R*_2_, respectively) as a function of the inverse of temperature. These contributions arise from the two ZARC elements present in the equivalent circuit shown in Figure 6a. Capacitance obtained from the fitting ranged from 1 × 10^−6^ to 5 × 10^−3^ F cm^−2^ for *C*_1_, with a characteristic frequency in the range of 10^4^–10^2^ Hz, and from 5 × 10^−4^ to 5 × 10^−2^ F cm^−2^ for *C*_2_, with a frequency within the range 10^1^–10^0^ Hz. Charge transfer phenomena are compatible with *C*_1_ [46,57,97,98], while the larger values of *C*_2_ are characteristic of surface exchange reactions such as the dissociation and adsorption of oxygen molecules [42,98,99]. In Supplementary Information (Section S3, Figure S3), the plots of *C*_1_ vs. *T* and *C*_2_ vs. *T* of the four cells are included for the sake of completeness. An observed increase in capacitance after infiltration (for both *C*_1_ and *C*_2_) is compatible with the increase in contact points between CGO and LSCF, as previously reported by dos Santos Gomez et al. for similar functionalization through infiltration [98]. Considering mesoporous CGO, this increase in the capacitance value was not observed for *C*_2_ (Figure S3b), which is probably related again to the charge transfer blocking effect of SiO_2_ impurities and dopant segregated to the surface of the mesoporous powder [100,101]. This hypothesis is compatible with *ASR*_pol_ behavior since *R*_1_ remains similar for 10-CGO and 10-CGO_meso_, which indicates a certain blocking effect of the charge transfer at the LSCF–CGO interfaces, while *R_2_* is notably improved for 10-CGO_meso_, which indicates better performance for non-charge transfer phenomena taking place at the surface.

## 4. Conclusions

Composites layers of LSCF–CGO were successfully fabricated by automatic infiltration using DOD-IJP for their use as functional oxygen electrodes in solid oxide cells. Symmetrical electrochemical cells with infiltrated oxygen electrode functional layers (YSZ/LSCF–CGO/LSCF) were fabricated and measured in the present work. After optimization of the formulated LSCF ink, infiltration of different pre-sintered CGO scaffolds was successfully demonstrated. The optimization of the saturation parameter of the ink was critical to ensure the homogeneous infiltration of the porous backbones. Symmetrical cells with optimal infiltrated layers showed a minimum *ASR* of ≈1.2 Ω cm^2^ at 750 °C, which is competitive with state-of-the-art results reported for composites of similar materials. Electrochemical impedance spectroscopy analysis carried out in this work concluded that optimization of the infiltration of the ceramic backbones reduces polarization resistance by improving the activity of the electrodes. Electrochemical characterization also highlighted the improvement in the serial contribution, due to better catalytic activity and current collection along the electrode. Moreover, CGO scaffolds fabricated by using mesoporous powders were successfully infiltrated, resulting in an even lower *ASR*_pol_, ≈0.18 Ω cm^2^ at 750 °C, due to an increase in the active area of the infiltrated backbone. Overall, this work confirms the recently reported good electrode infiltration in solid oxide cells and paves the way for automation of reproducible and scalable large-area infiltration based on ink jet printing technology.

## Figures and Tables

**Table 1:** Characteristics of the cells produced. Commercial YSZ tape (≈150 μm) was used for all the fabricated cells. A barrier layer made of commercial CGO decorated by Co oxide was deposited and sintered at 1275 °C. Commercial CGO scaffolds were sintered at 1250 °C and the mesoporous CGO at 900 °C.

CELL	Scaffold	Infiltration	Saturation
CGO	Commercial CGO	No	-
5-CGO	Commercial CGO	Yes	5/20 sat.
10-CGO	Commercial CGO	Yes	10/20 sat.
10-CGO_meso_	Mesoporous CGO	Yes	10/20 sat.

## Data Availability

Not applicable.

## Supplementary Materials

The following are available online at https://www.mdpi.com/article/10.3390/nano11123435/s1, Figure S1: Printing system form Print3D Solutions with a HP C6602A commercial cartridge. Figure S2: Characterization of the mesoporous CGO powder after synthesis and chemical etching by NaOH solution to remove the silica template. SEM micrographs a two different magnifications (**a**) and (**b**). Pore area measured by BET analysis (**c**) and XRD characterization of the powders (**d**). Figure S3: Capacitance values of *R*_1_ and *R*_2_ elements used for the fitting of the EIS spectra represented as function of temperature, here presented as *C*_1_ (**a**) and *C*_2_ (**b**).
