# Supplementary material for: Solid Oxide Cell Electrode Nanocomposites Fabricated by Inkjet Printing Infiltration of Ceria Scaffolds
Source: Nanomaterials (Basel). 2021 Dec 18;11(12):3435. doi: 10.3390/nano11123435 (PMC8704428; doi:10.3390/nano11123435)
Supplement: Supplementary file 1 [file nanomaterials-11-03435-s001.zip › nanomaterials-1442406-supplementary.pdf]

nanomaterials

MDPI

Supplementary information

Solid Oxide Cell Electrode Nanocomposites Fabricated by Inkjet Printing Infiltration of Ceria Scaffolds

Simone Anelli [1], Luis Moreno-Sanabria [2], Federico Baiutti [1], Marc Torrell [1] and Albert Tarancón [1,3,*]

[1] Catalonia Institute for Energy Research (IREC), Department of Advanced Materials for Energy, Jardins de les Dones de Negre, 1, Sant Adrià de Besòs, 08930 Barcelona, Spain; sanelli@irec.cat (S.A.); fbaiutti@irec.cat (F.B.); mtorrell@irec.cat (M.T.)
[2] Institute of Ceramics and Glass (ICV-CSIC), Kelsen 5, 28049 Madrid, Spain; l.moreno@icv.csic.es
[3] ICREA, Passeig Lluís Companys 23, 08010 Barcelona, Spain
* Correspondence: atarancon@irec.cat

Citation: Anelli, S.; Moreno-Sanabria, L.; Baiutti, F.; Torrell, M.; Tarancón, A. Solid Oxide Cell Electrode Nanocomposites Fabricated by Inkjet Printing Infiltration of Ceria Scaffolds. *Nanomaterials* **2021**, *11*, 3435. https://doi.org/10.3390/nano11123435

Academic Editors: Rumen I. Tomov and Graeme Watson

Received: 14 October 2021
Accepted: 2 December 2021
Published: 18 December 2021

Publisher's Note: MDPI stays neutral with regard to jurisdictional claims in published maps and institutional affiliations.

Copyright: © 2021 by the authors. Licensee MDPI, Basel, Switzerland. This article is an open access article distributed under the terms and conditions of the Creative Commons Attribution (CC BY) license (http://creativecommons.org/licenses/by/4.0/).

S1. Printing System

Figure S1 shows the custom made inkjet printer produced by Print3d Solutions (02071 Albacete, Spain). One can notice the commercial cartridge C6602A from Hewlett-Packard (Palo Alto, CA 94304, USA) used for the deposition process. Such a cartridge has twelve nozzles with a diameter ≈60 µm [1]. The movements of the 3-axis system are controlled by Arduino (https://www.arduino.cc/, webpage consulted the 13-9-2021) and the printing process (with the C6602A cartridge) by Processing© software (version 3.1.1, Processing Foundation, Brooklyn, NY 11201, USA). The printing system allows to modify the speed of the layer deposition, therefore the saturation parameter of the ink.

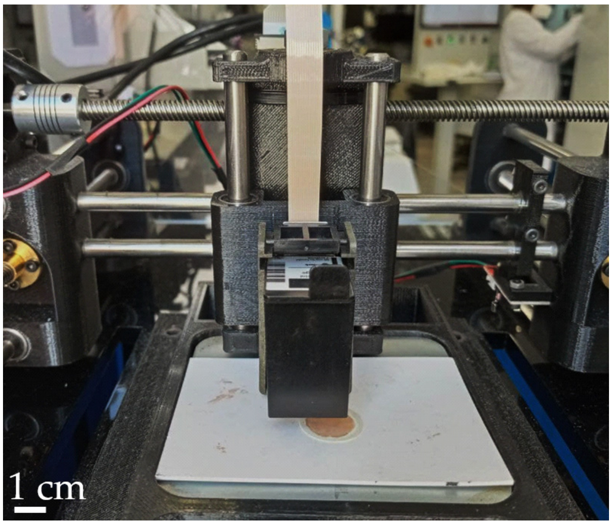

Figure S1. Printing system form Print3D Solutions with the HP C6602A commercial cartridge.

S2. Mesoporous Powder Characterization

For the fabrication of the 10-CGO_{meso} cell, mesoporous powders synthetized by hard-template method were synthetized and characterized by an Auriga scanning electron microscope (SEM—Carl ZEISS, 73447 Oberkochen, Germany) and X-ray diffraction (XRD—Bruker-D8 Advance equipment, Billerica, MA 01821, USA) in order to corroborate the presence of the mesoporous structure and its periodicity. In particular the SEM micrographs of Figure S2a,b highlight the presence of the bi-continuous structure provided by the silica hard template with *Ia3d* symmetry [2,3]. Figure S2c shows the desorption incremental pore area distribution obtained by Tristar II Brunauer–Emmett–Teller (BET, Micromeritics, Norcross, GA 30093, USA) analysis. Two peaks around ≈3 and ≈10 nm are

shown, demonstrating the presence of both the two possible structures which can be obtained by the complete or partial filling of the bi-continuous channels of the KIT-6 template [4–6]. The simultaneous presence of both structure is the most common situation after synthesis [7]. The BET area value measured was ≈44.7 m^2 g^{-1}.

In Figure S2d one can observe the XRD pattern of the powders, which presents the typical peaks of the CGO fluorite structure. This characterization confirmed the success of the synthesis process of the CGO mesoporous powders. These powders were used as backbone layer for the 10-CGO_{meso} cell, with the aim to combine the automatic inkjet infiltration with the advantages in terms of exposed surface area of the mesoporous CGO.

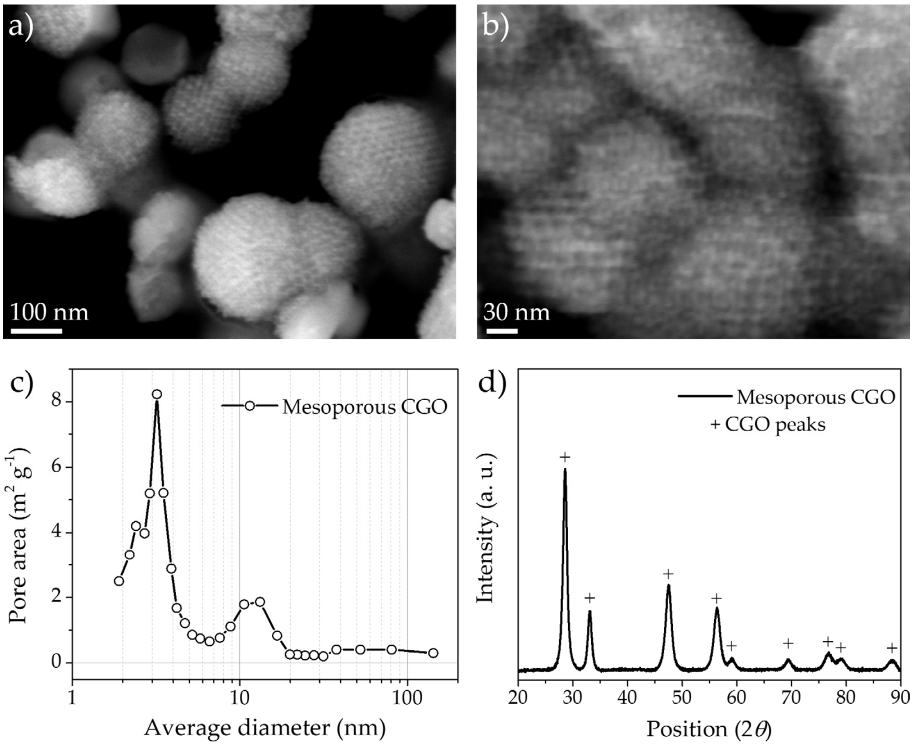

Figure S2. Characterization of the mesoporous CGO powder after synthesis and chemical etching by NaOH solution to remove the silica template. SEM micrographs a two different magnifications (**a**) and (**b**). Pore area measured by BET analysis (**c**) and XRD characterization of the powders (**d**).

S3. Capacitances of R_1 and R_2 Contributions

For a more detailed analysis of the different detected contributions, Figure S3 presents the capacitance values of the two ZARC elements for each cell. In particular, Figure S3a and Figure S3b present the relative capacitance values, C_1 vs. T and C_2 vs. T. The capacitance values of the $R_{p1}C_1$ element (with a frequency range between 10^4–10^2 Hz) were comprised between $1{\cdot}10^{-6}$–$5{\cdot}10^{-3}$ F cm^{-2}. Similar contributions for LSCF-CGO electrodes are consistent with charge transfer phenomena [8–11]. The $R_{p2}C_2$ element (10^1–10^0 Hz), was characterized by larger capacitance values $5{\cdot}10^{-4}$–$5{\cdot}10^{-2}$ F cm^{-2}. These frequency and capacitance values are identified with surface exchange reaction like dissociation and adsorption of oxygen molecules [10,12,13].

Dos Santos Gomez *et al.* states that electrode functionalization through infiltration can increase capacitance values for both processes due to the increment of contact points between CGO and LSCF [10]. This was also revealed in the present study for the higher frequency contribution (C_1). When considering C_2, this increment of the capacitance value was not observed for the 10-CGO_{meso} cell (Figure S3b). This effect was probably correlated to SiO_2 contamination which is due to the synthesis process of the mesoporous powder [14]. The contamination played a role reducing the contact point between CGO and infiltrated LSCF [15].

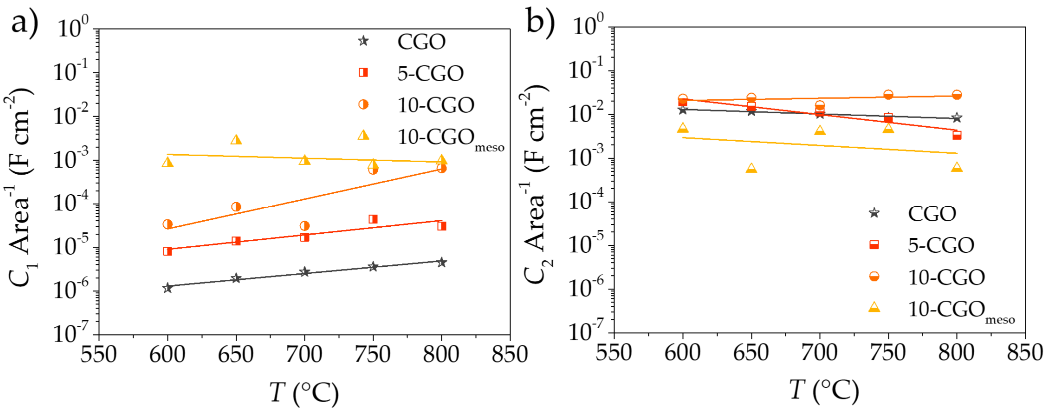

Figure S3. Capacitance values of R_1 and R_2 elements used for the fitting of the EIS spectra represented as function of temperature, here presented as C_1 (**a**) and C_2 (**b**).

References

1. Tyler, H. Open Source Possibilities for Inkjet 3D Printing, 2014. Advanced Manufacturing Research Institute, Rice University. Available online: https://www.scribd.com/document/414152953/Open-Source-Possibilities-for-Inkjet-3D-Printing-Advanced-Manufacturing-Research-Institute-Rice-University-Harrison-Tyler-2014 (accessed on 14 September 2021).
2. Doi, Y.; Takai, A.; Sakamoto, Y.; Terasaki, O.; Yamauchi, Y.; Kuroda, K. Tailored synthesis of mesoporous platinum replicas using double gyroid mesoporous silica (KIT-6) with different pore diameters via vapor infiltration of a reducing agent. *Chem. Commun.* **2010**, *46*, 6365-6367. https://doi.org/10.1039/C0CC01196G.
3. Burns, G.; Glazer, A.M.; Maradudin, A.A. Space Groups for Solid State Scientists. *Phys. Today* **1979**, *32*, 51, https://doi.org/10.1063/1.2995619.
4. Ye, Y.; Jo, C.; Jeong, I.; Lee, J. Functional mesoporous materials for energy applications: solar cells, fuel cells, and batteries. *Nanoscale* **2013**, *13*, 4584-4605. https://doi.org/10.1039/C3NR00176H.
5. Zhang, L.; Jin, L.; Liu, B.; He, J. Templated Growth of Crystalline Mesoporous Materials: From Soft/Hard Templates to Colloidal Templates. *Front. Chem.* **2019**, *7*, 2296-2646. https://doi.org/10.3389/fchem.2019.00022.
6. Anelli, S. Advanced strategies for Solid Oxide Electrolysis cells. Ph.D. Thesis, Universitat Autònoma de Barcelona, Barcelona, Spain, 2021.
7. Ren, Y.; Ma, Z.; Bruce, P. G. Ordered mesoporous metal oxides: synthesis and applications. *Chem. Soc. Rev.* **2012**, *41*, 4909. https://doi.org/10.1039/C2CS35086F.
8. Baumann, F.S.; Fleig, J.; Habermeier, H.-U.; Maier, J. Impedance spectroscopic study on well-defined (La,Sr)(Co,Fe)$O_{3-\delta}$ model electrodes. *Solid State Ionics* **2006**, *177*, 1071-1081. https://doi.org/10.1016/j.ssi.2006.02.045.
9. Burye, T.E.; Nicholas, J.D. Nano-ceria pre-infiltration improves $La_{0.6}Sr_{0.4}Co_{0.8}Fe_{0.2}O_{3-x}$ infiltrated Solid Oxide Fuel Cell cathode performance. *J. Power Sources* **2015**, *300*, 402-412. https://doi.org/10.1016/j.jpowsour.2015.09.080.
10. dos Santos-Gómez, L.; Porras-Vázquez, J.M.; Losilla, E.R.; Martín, F.; Ramos-Barrado, J.R.; Marrero-Lopez, D. Stability and performance of $La_{0.6}Sr_{0.4}Co_{0.2}Fe_{0.8}O_{3-\delta}$ nanostructured cathodes with $Ce_{0.8}Gd_{0.2}O_{1.9}$ surface coating. *J. Power Sources* **2017**, *347*, 178-185. https://doi.org/10.1016/j.jpowsour.2017.02.045.
11. Tomov, R.I.; Mitchel-Williams, T.B.; Maher, R.; Kerherve, G.; Cohen, L.; Payne, D.J.; Kumar, R.V.; Glowacki, B.A. The synergistic effect of cobalt oxide and Gd-CeO_2 dual infiltration in LSCF/CGO cathodes. *J. Mater. Chem. A* **2018**, *6*, 5071-5081. https://doi.org/10.1039/C7TA10990C.
12. Xu, X.; Jiang, Z.; Fan, X.; Xia, C. LSM–SDC electrodes fabricated with an ion-impregnating process for SOFCs with doped ceria electrolytes. *Solid State Ionics* **2006**, *177*, 2113–2117, https://doi.org/10.1016/j.ssi.2006.01.043.
13. Nie, L.; Liu, M.; Zhang, Y.; Liu, M. $La_{0.6}Sr_{0.4}Co_{0.2}Fe{+}O_{3-\delta}$ cathodes infiltrated with samarium-doped cerium oxide for solid oxide fuel cells. *J. Power Sources* **2010**, *195*, 4704-4708. https://doi.org/10.1016/j.jpowsour.2010.02.049.
14. Ghom, S.A.; Zamani, C.; Nazarpour, S.; Andreu, T.; Morante, J. Oxygen sensing with mesoporous ceria–zirconia solid solutions. *Sensors Actuators B: Chem.* **2009**, *140*, 216–221, https://doi.org/10.1016/j.snb.2009.02.078.
15. Druce, J.; Kilner, J.A. Improvement of Oxygen Surface Exchange Kinetics for CGO with Surface Treatment. *J. Electrochem. Soc.* **2013**, *161*, F99–F104, https://doi.org/10.1149/2.049401jes.
